# The effect of steel waste addition as a cement replacement on the mechanical and radiation shielding properties of sustainable concrete

**DOI:** 10.1038/s41598-026-51323-2

**Published:** 2026-05-13

**Authors:** Shymaa Mukhtar, Hossam El-Din M. Sallam, Rasha A. Elsadany

**Affiliations:** 1 Civil Engineering Department, Higher Technological Institute (HTI), 10th of Ramadan City, 44629 Egypt; 2 Materials Engineering Department, Faculty of Engineering, Zagazig University, Zagazig, 44519 Egypt; 3 Radiation Engineering Department, National Center for Radiation Research and Technology, Egyptian Atomic Energy Authority, Cairo, Egypt

**Keywords:** Sustainable concrete, Steel waste materials, Mechanical properties, Attenuation coefficient, Engineering, Environmental sciences, Materials science

## Abstract

Replacing cement with steel additives (waste materials or steel ore (iron oxide)) contributes to the sustainable production of concrete, improving its mechanical and radiation-shielding properties. Consequently, an experimental investigation was undertaken to examine the effects of using waste steel additives — such as brake lining, rolling mill scales, iron filings, and steel ore (hematite) — on the properties of sustainable concrete containing slag as a coarse aggregate. The effect of varying ratios of steel additives on the physical, mechanical, and gamma radiation shielding properties of sustainable concrete was examined. Gamma-attenuation measurements were taken with these mixtures at three gamma energies: 0.66, 1.17, and 1.33 MeV. The experimental results showed that using brake lining, rolling mill scales, iron filling, and hematite improved the physical, mechanical, and radiation properties of concrete. SEM analysis indicates microstructural improvement with waste steel. It was observed that the linear attenuation coefficient lies between 0.0923 cm^-1^ and 0.2532 cm^-1^. The study concluded that adding steel additives to the concrete mixture delivered superior performance, provided a solution for waste disposal, and promoted environmental sustainability.

## Introduction

In recent decades, the concrete industry has demonstrated that encouraging recycling is a viable path to sustainable growth. This reduces the use of natural resources and promotes the use of recycled materials instead of disposing of them in landfills^1^. Furthermore, the sustainability objective, which is the driving force behind the majority of today’s social challenges, requires greater recycling of waste and by-products, particularly in high-impact industries such as the cement and concrete sectors^2^. Slag, steel mill scale, iron filler, and other solid waste products are produced along with iron and steel. Slag can be used to make concrete that hardens rapidly by accelerating the cement hydration process^3^. When utilized as a binder in concrete, the SiO_2_, Al_2_O_3_, and calcium oxides found in steel sludge and steel slag improve compressive and flexural strength^4^. Cement hydration is aided by the higher concentration of active forms of dicalcium and tricalcium silicate found in steel slag. Therefore, using steel slag as a replacement for sand in concrete results in higher compressive strength than normal concrete^5^. When using slag as a replacement for coarse aggregate (ranging from 0% to 100%) and three concrete compressive strength levels (20, 30, and 40 MPa), the compressive strength increased with a 25% replacement of natural coarse aggregate. The splitting tensile strength reached its optimum value at 25% natural aggregate replacement^6^. However, the mixture with 30% steel slag replacement of coarse aggregate exhibited favorable strength properties and workability^7^. Concrete made from industrial waste iron and steel has the potential to serve as a shield against gamma radiation^8^. As a by-product of the steelmaking industry, millions of tons of steel mill scale (approximately 35–40 kg/t) are produced. To protect the environment, these steel mill scales must be handled, which requires developing a new method to deliver high-quality construction materials. The steel mill scale was generated during hot-rolling of steel^9^. Using pulverized steel mill scale as a partial replacement for cement in concrete enhances the pozzolanic action, as it contains silicate^10^. When 40% of the fine aggregate was replaced with mill scale, strength increased, and workability decreased^11^. However, using steel mill scale recycling as a partial substitute for fine aggregate provides a workable, economical, and sustainable option at a 60% replacement ^12^. Using iron fillers as a complete replacement for sand resulted in a substantial increase in compressive and tensile strength and a decrease in abrasion in concrete^13^. However, when iron powder was used as a partial replacement for portland cement, compressive and tensile strength increased by 2.5% and 1.5%, respectively. As the percentage of iron powder increased, the workability and porosity decreased^14^. When adding iron to concrete at 10%, 20%, and 30%, the compressive strength increased gradually, while the tensile strength was only slightly affected when the iron filings exceeded 10%^15^. Different percentages of iron filings (0.5%, 0.75%, and 1%) were added to three sustainable concrete types: geopolymer concrete with natural aggregates, traditional concrete with natural aggregates, and traditional concrete with recycled aggregates. Results indicate that increasing the amount of iron filings to 1.0% improved the percentage of indirect tensile strength, flexural strength, compressive strength, SEM, and modulus of elasticity^16^.

Brake lining waste from heavy vehicles, which may contain metallic and carbon-based materials, was used in mortar to increase its electrical conductivity; up to 70% of the waste was used as a replacement for sand. The results showed that the mortars containing brake lining waste had lower impedance (indicating a trend toward higher electrical conductivity) and lower compressive strength^17^. It is possible to use two different types of waste brake shoes, ie, pyrolysis-friction reclaimed materials of waste brake-shoe (P-FRMWBS) and mechanical grinding friction reclaimed material of waste brake-shoe (G-FRMWBS) as a replacement for fine aggregate. The results indicated that when the replacement rate was 5%, G-FRMWBS increased mortar strength by 16.6% and 17.5%; when the replacement rate was 10%, the increase was 19.2% and 19.2%^18^. Milasi et al.^19^ used barite, hematite, and lead powders as replacements for cement at 10%, 15%, 20%, 25%, and 30% in ultra-high-performance concrete (UHPC), resulting in reduced compressive and flexural strengths. The highest compressive strengths for barite and hematite were at 10% cement content. Among all tested mixes, the highest attenuation and lowest half-life coefficients were recorded for UHPC with 30% lead powder (0.23 cm^-1^ and 3 cm, respectively). This was about 53% higher than that of the lead-free UHPC, and its half-life coefficient was 35% lower.

Radiation poses a major risk to both structural integrity and human health. It encompasses both ionizing and non-ionizing forms of radiation. Non-ionizing radiation includes electromagnetic waves with wavelengths of 10 nm or longer, while ionizing radiation comprises X-rays, γ-rays, and subatomic particles and is divided into direct and indirect ionizing radiation. The main concern regarding ionizing radiation, especially gamma rays and neutrons, is their high penetration power and long range, which can pose risks to people and buildings. Materials with high density, including slag, hematite, barite, steel bismuth, and lead, are recommended for shielding against gamma radiation due to their effectiveness. For shielding against neutrons, materials rich in hydrogen are often combined with high atomic-number elements and thermal-neutron absorbers, referred to as neutron poisons^20^. In recent decades, researchers have widely investigated various concrete constituents to develop shielding structures with high physical and mechanical performance, ensuring strong protection against gamma rays and neutrons^21–23^. For instance, the effectiveness of nuclear radiation shielding barriers incorporating various plaster constituents, namely, titanium, barite, steel slag, and hematite powders, has been examined by Ali et al^21^. The results demonstrated that steel slag–titanium plaster is a suitable option due to its enhanced radiation shielding performance. It also satisfies the criteria for sustainable design^21^. Furthermore, Elabbasy et al^22^ conducted a systematic evaluation of the mechanical properties and radiation-shielding performance of different UHPC mixtures incorporating individual and hybrid combinations of steel, polyvinyl alcohol, polypropylene, and natural jute fibers. The results showed that the UHPC mix reinforced with steel fibers alone achieved the highest mechanical performance, with a compressive strength of 140 MPa at 28 days. Incorporating hybrid fibers (steel fibers with other fibers) reduced compressive, tensile, and flexural strengths to varying degrees. Despite this reduction, hybrid fiber mixes still provided acceptable γ-ray shielding performance, with added benefits such as improved cost efficiency, flexibility, and sustainability^22^.

Azzam et al^23^ investigated the impact of partially replacing cement with 10% and 20% rice husk ash (RHA) and sugarcane bagasse ash (SCBA) on the physical properties, mechanical performance, microstructural characteristics, and radiation-shielding capabilities of high-strength concrete (HSC). The results indicated that the pozzolanic activity of RHA and SCBA improved hydration and microstructure, and reduced setting time, thereby enhancing mechanical properties. Radiation shielding simulations showed that concrete containing SCBA provided better γ-ray attenuation than RHA-based samples. Additionally, the 20% SCBA mixture exhibited the highest fast-neutron removal cross-section among all tested specimens^23^. Additionally, the impact of incorporating blended dealuminated metakaolin (DK), limestone (LS), and silica fume (SF) powders as partial replacements for cement on the physical, mechanical, and radiation properties of HSC was investigated. Monte Carlo simulations and Phy-X software were employed to assess the radiation shielding characteristics of the produced concretes against γ-rays and fast neutrons. Results revealed that the inclusion of blended SF, LS, and DK powders in HSC lowered its workability, whereas compressive and tensile strengths were increased. The γ-ray attenuation analysis indicated a modest gain in shielding effectiveness for HSC, particularly in DK10 and SF15LS10DK10 mixes, which achieved the highest linear attenuation coefficients (LACs)^24^. On the other hand, the effects of ambient and elevated temperatures on the LAC values of concrete mixes incorporating lead monoxide, nanomagnetite, and nanogranodiorite. Results revealed that elevated temperatures caused a substantial decline in the LAC of the control mix, notably at 800 °C; however, nanomaterial incorporation helped maintain improved shielding efficiency^25^.

Mostafa et al^26^ investigated the effects of ferrosilicon and aluminum nanowastes on lightweight geopolymer bricks, highlighting their potential for radiation shielding applications. Simulations using the Monte Carlo method showed that ferrosilicon waste bricks exhibited greater attenuation of gamma rays and neutrons than their aluminum counterparts^26^. For sustainability. Abouelnour explored the feasibility of utilizing Heavy Silicate Minerals (HSMs) as an eco-friendly volumetric replacement for natural sand in concrete. They assessed the concrete’s workability and density, compressive and tensile strengths, microstructure via XRD, SEM, and EDX, and its radiation shielding capabilities. An increase in HSMs led to a considerable reduction in concrete workability and to improvements in density, compressive strength, and tensile strength. Gamma-ray LACs for the examined concrete mixes showed a distinct upward trend: CO < 25HSMs < 50HSMs < 75HSMs < 100HSMs^27^. Furthermore, Mostafa et al^28^ investigated how basil plant ash (BPA) influences the mechanical properties, durability, radiation shielding effectiveness, environmental impact, and economic viability of UHPC. Radiation protection performance against gamma rays and neutrons was assessed using Monte Carlo simulations and Phy-X software. The main results of this study indicate that, under normal conditions, the BPA mixes showed improved mechanical performance, greater resistance to aggressive environments, reduced cement content, and decreased CO2 emissions.• BPA-based UHPC provided improved protection against γ-rays and fast neutrons, consistently exceeding the performance of control (CO) mixes under varying environmental conditions^28^. In addition, Mahmoud et al^29^ evaluated the effect of rice husk–derived biochar (RHB) on concrete properties, including its physical, mechanical, and radiation-shielding performance. It was observed that RHB led to a longer setting time and improved the mechanical properties of cement. The gamma-ray attenuation results showed a modest enhancement in shielding performance, with the 15% RHB sample achieving the highest LAC due to its higher density, while the 25% RHB sample was less effective.

The feasibility and novelty of the present work lie in the use of waste iron as a sustainable replacement for cement in slag-based concrete, where slag serves as the coarse aggregate. A thorough methodological strategy was used to assess the effects of integrating waste iron on slag-based concrete characteristics, including physical, mechanical, and microstructural evaluations, and to determine crucial radiation protection metrics (e.g., LAC, half-value thickness (HVT), and tenth-value thickness (TVT). These calculations yielded new insights into the gamma-ray attenuation properties of slag-based concrete modified with waste iron.

## Experimental work

### Constituent materials and mix proportions

**Fig. 1:**
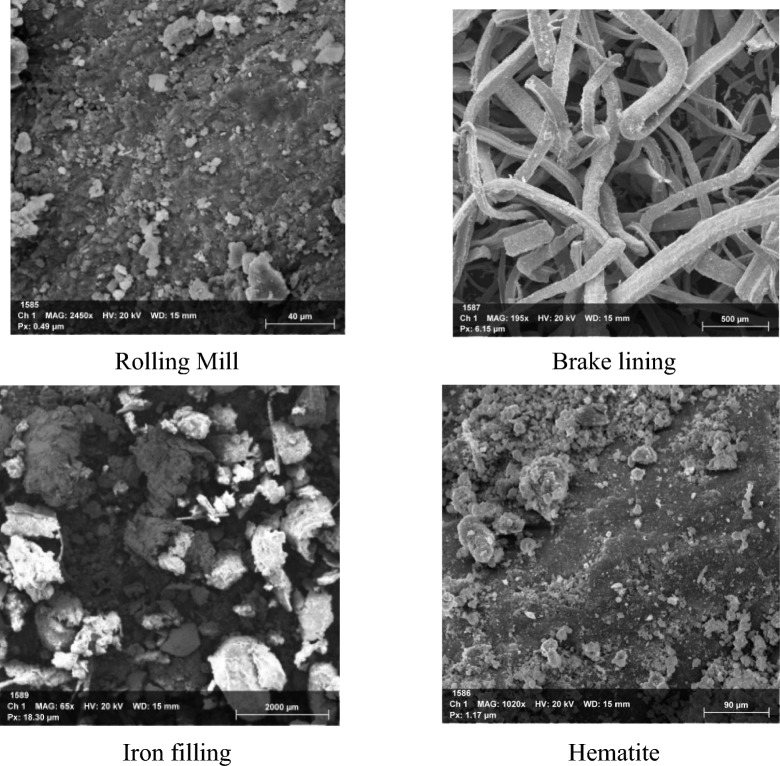
SEM of additives.

Materials used in the paper are sulfate-resistant cement (SRC) – SRC 52.5N, additives such as brake lining (B), rolling mill scale (R), hematite powder (H), iron filings (I), slag as a coarse aggregate, sand as a fine aggregate, water, and superplasticizer. The used mill steel scale was produced during hot rolling at Helwan Iron and Steel Factory. However, the brake linings used came from trucks and other large vehicles and were acquired from a mechanical workshop in 10^th^ Ramadan City. Additionally, the iron filler that was used came from the Helwan Iron and Steel Factory in Egypt. Hematite is the mineral form of iron oxide that has crystallized in a rhombic lattice system with a chemical formula of Fe_2_O_3_. The physical and mechanical properties of the SRC and additives are shown in Table 1, and the SEM photos of them are shown in Fig 1.

**Table 1:** Physical and mechanical properties of SRC and additives.

Properties	Rolling mill (R)	Brake lining (B)	Iron filling (I)	Hematite (H)	Cement (SRC)
Color	Black	Gray	Grayish	Brown	Gray
Specific gravity	3	3.1	3.62	5.00	3.15
Porosity %	40	42	41	11	20
Water absorption	0.5%	0.7%	0.8%	3%	6%
Bulk density kg/m^3^	2000	2100	2900	4300	1440

The superplasticizers are Sikamen–NN (high-range water reducing concrete admixture from Sika company, 0.6-3% by weight of cement). Electric arc furnace steel slag (supplied by Beshay Steel Group, Egypt) was used as coarse aggregates in concrete. The slag, with a specific gravity of 3.5 and a density of 1800 kg/m^3^, was crushed and sieved to produce a particle size distribution similar to that of natural coarse aggregate, according to ESS 1109/2002^30^. Max nominal size of slag 10 mm, fineness modulus 7, Impact coefficient 10%, Los Angeles-abrasion coefficient 18.5%, and crushing coefficient 22%. Quartz sand was used as the fine aggregate, with a maximum particle diameter of 2.4 mm and a fineness modulus of 2.31.

The mix design for 13 mixes was as follows: cement/cementitious materials of 400 kg/m^3^, slag of 1245 kg, sand of 830 kg, water/cement (W/C) ratio of 0.4, and superplasticizer of 2%. The additives mentioned above (B, R, H, and I) were added as cement weight replacement by three different ratios: 10%, 20%, and 30%. Table 2 explains the code of these mixes. The slump of each mix was measured immediately after mixing, in accordance with the Egyptian Standard Specifications ESS 1658/2006^31^. All test specimens were removed from their molds 24 hours after casting and then saturated in water tanks for 7 or 28 days, depending on the test age.

**Table 2:** The proportions of the cementitious materials of the thirteen mixes used.

Mix	Cement kg/m	Additives kg/m^3^
Control	400	0
B10	360	40 brake lining
B20	320	80 brake lining
B30	280	120 brake lining
R10	360	40 rolling mill scale
R20	320	80 rolling mill scale
R30	280	120 rolling mill scale
H10	360	40 hematite powder
H 20	320	80 hematite powder
H 30	280	120 hematite powder
I 10	360	40 iron filings
I 20	320	80 iron filings
I 30	280	120 iron filings

### Mechanical and radiation shielding concrete properties, and microstructure evaluation

Tests on hardened concrete were conducted at 28 days of age as follows: (a) The compressive strength of different mixes was measured by testing 150 mm-side-length cubes, and indirect tensile strength was measured by testing 300 mm-high, 150 mm-diameter cylinders in accordance with the Egyptian Standard Specifications ESS 1658/2006^31^. The average value from three tested specimens for each mix was taken as the representative strength. The specimens were demolded 24h after casting and cured for 28 days before testing. (b) The density measurement was performed in accordance with ASTM C642-97^32^.

**Fig. 2:**
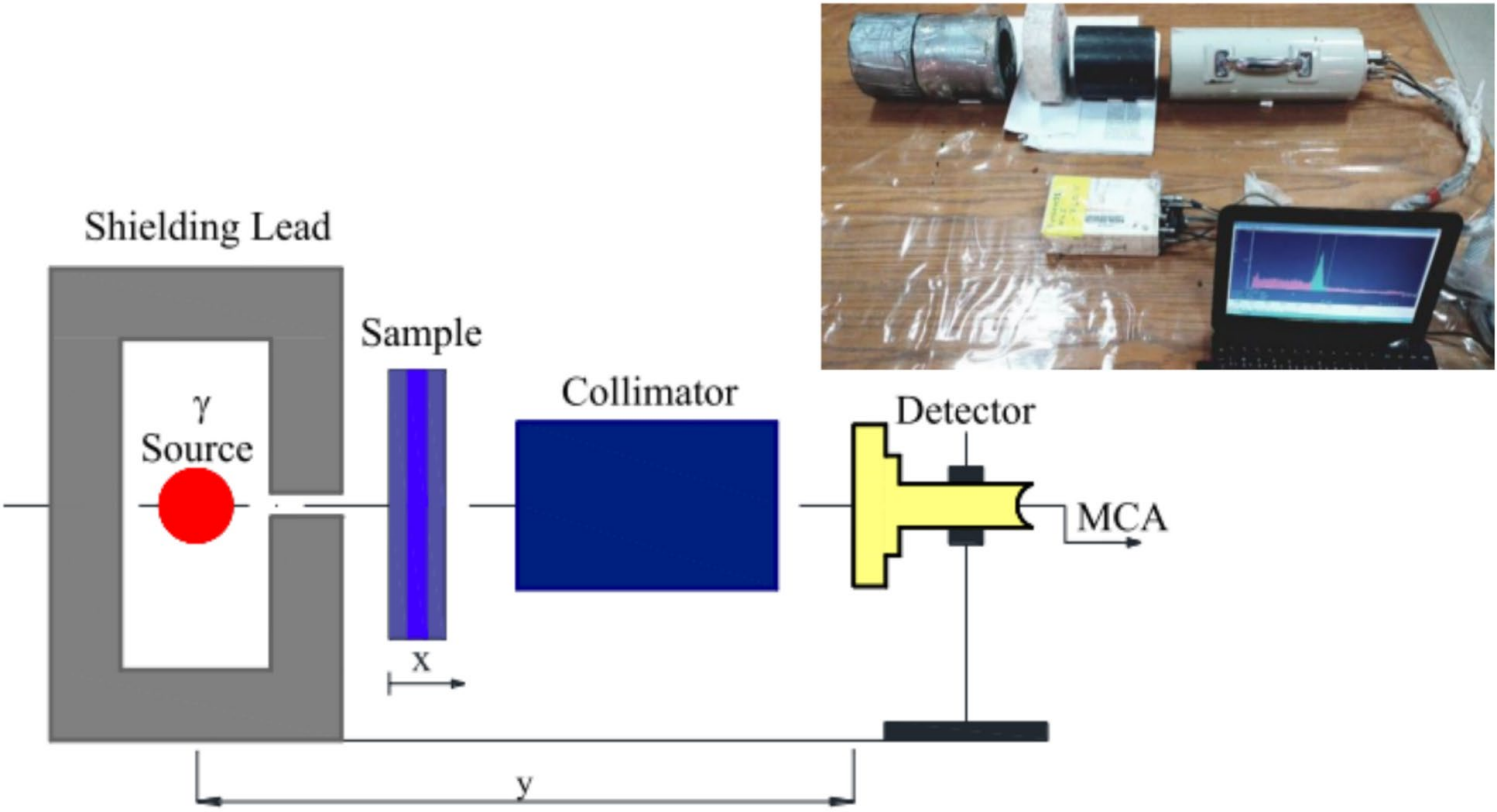
Set up of the radiation attenuation test.

Gamma-ray shielding experiments were conducted at the National Center for Radiation Research and Technology, a division of the Egyptian Atomic Energy Authority in Cairo, Egypt. In these experiments, a Co-60 or Cs-137 point source with an activity of ten microcurie (µCi) was used. Samples shaped as half circles with a 15 cm diameter, 7 cm width, and different thicknesses were used in the tests. A highly sensitive NaI detector was used to accurately measure gamma activity. It was strategically placed behind the shield and seamlessly connected to a sophisticated, computerized multichannel analyzer (MCA), as shown in Fig 2. This setup ensures precise, reliable readings, thereby enhancing the effectiveness of our analysis. Using the MCA, peak curves of the source materials were obtained at multiple thickness levels. A cylindrical lead shield encloses the Cs-137, which emits a characteristic, strong gamma-ray photon at 0.662 MeV, or Cobalt-60, a high-energy radioisotope that emits two primary gamma rays at 1.17 MeV and 1.33 MeV, directing radiation through a small window for narrow-beam exposure. The specimens were precisely aligned with the detector and placed between the collimator and the gamma-ray source. The distance Y in Fig 2 was 20 cm, and the time of testing was about 15 min. The photon energy was measured at 0.66, 1.17, and 1.332 MeV, and backscattering from the room was reduced by using insulators.

Radiation intensity is reduced to safe levels through a phenomenon known as attenuation, which is quantified by the attenuation coefficient. The coefficient µ is highly significant in radiation protection studies and is expressed in units of cm⁻1. It can be calculated from equation 1.

1 $$I = I_{o} e^{ - \, \mu x}$$

Where I = intensity of radiation at point (x), Io: Intensity of the source, µ: absorption coefficient of absorber material, "attenuation coefficient. When the absorber thickness is just enough to reduce the intensity by a factor of 1/2 or 1/10, the corresponding thicknesses are called the HVT and TVT, respectively, and are given in inches or centimeters. It is determined from:

2 $$T_{1/2} = \, 0.693/ \, \mu$$

3 $$T_{1/10} = \, 2.3026/ \, \mu$$

The type and energy of the radiation, as well as the absorbed material, affect these values. Relaxation Length (Tr), Tr = 1/µ, is another quantity that resembles the half-value. Tr represents a 63.2 percent reduction in thickness, similar to the 50 percent reduction in the original thickness.

Backscattered electron (BSE) images from SEM were obtained to examine the microstructure and provide visual evidence of the microstructural components that enhance mechanical and physical properties. Samples were examined morphologically using a scanning electron microscope model ZEISS-EVO 15-UK operating at 25 kV. After the strength test, the small specimen containing the solidified cement and waste steel was removed from the crushed specimen, dried for a day at 50 degrees Celsius, and then coated with a thin layer of gold to prepare it for microstructural analysis. SEM images were taken at the same magnification (200 kX) for use in the ImageJ program. By detecting grey tones in image-processing software, BSE images help predict the presence of pores and hydrated zones in the specimens. The BSE images, with dimensions of 1024x943, contained 256 levels of grey, ranging from 0 (black) to 256 (white). The ImageJ program was used to process the SEM images using integration software and grey color histograms that show the cumulative grey color for each color degree.

**Fig. 3:**
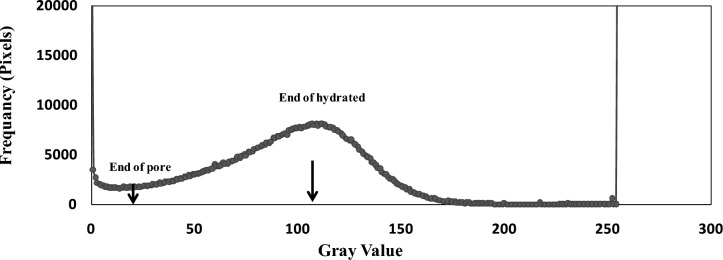
A histogram representing the frequency of gray color

The BSE images provide a powerful tool for accurately predicting the presence of pores and hydrated regions within specimens. The BSE images predict pores and hydrated regions in specimens by identifying gray levels in ImageJ. This software was utilized to process the SEM photos using grey. Visual histograms illustrate the cumulative gray intensity for each color level, along with preset thresholds and the associated integration software. Calculations were performed to determine the percentages of pores and hydrated portions in the examined specimens. Using the histogram’s lowest value from the first drop, the pores can be calculated, as described in^33,34^ (see Fig 3). The percentage of pores will be determined either by using a threshold at this point’s grey level or by integrating until this point’s frequency reaches a specified threshold.

**Fig. 4:**
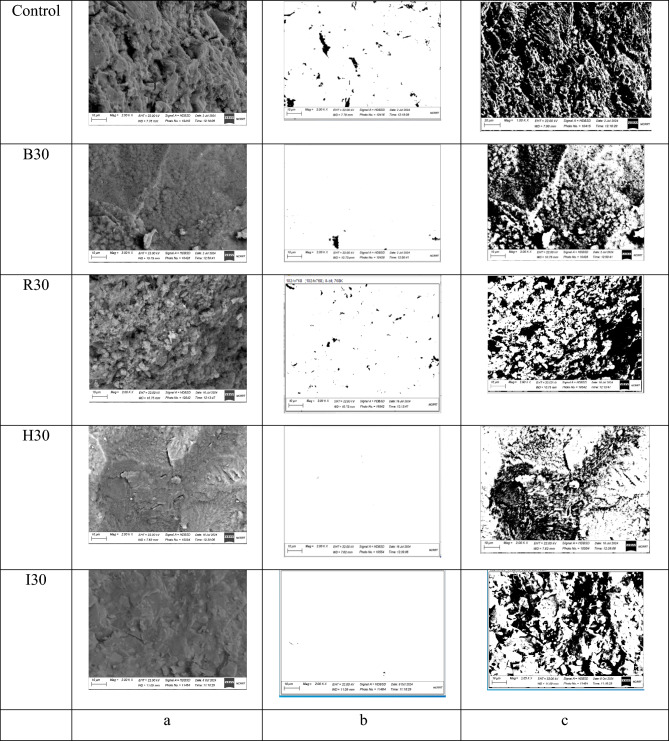
**a**) BSE images from the SEM technique for selected samples at 28 days, i.e., control B, R, H, and I samples, and the maximum percentage of waste materials, **b**) pores from the Threshold, and **c**) Cumulative pores and hydrated patches from the Threshold of the same images.

The histogram’s maximum value, as illustrated in Fig 3, indicates the integration limit for the hydrated particles of the specimens, including the pore thresholds. To determine the hydrated portions, the percentage of pores was subtracted from the integration value up to the maximum. As illustrated in Fig 4, the initial BSE image was obtained from the SEM test on a 28-day-old concrete specimen. In Figure 4b, a typical pore is shown using the threshold grey value obtained from the control and from histograms of some specimens (Fig 3). In Figure 4c the image of the same specimens as in Figure 4-a is processed using the grey color value corresponding to the histogram’s peak frequency. These investigations were carried out at the National Center for Radiation Research and Technology, Atomic Energy Authority, Cairo, Egypt**.**

## Results and discussion

### Results of fresh and hardened concrete

This section presents results for fresh and hardened concrete from different mixes, including slump, compressive, and tensile strengths, as listed in Table 3. The values shown in the Table represent the average of three measurements. A reduction in slump values was observed in all additive mixes compared with the control mix. It is worth noting that all the slump test results showed true slump shape. The percentage of reduction was 30%, 40%, 30%, 40%,30%, 20%, 50%, 40%, 40%, 10%,20% and 20% for B10, B20, B30, R10, R20, R30, H10, H20, H30, I10, I20 and I30, respectively. This reduction may be attributed to the increase in density in mixes with additives.

**Table 3:** Results of fresh and hardened concrete.

Mix			Compressive Strength (MPa)		Tensile strength (MPa)
	Slump (cm)	Density (kg/m^3^)	7days	28 days	
Control	10	2300	17.21	35.75	4.6
(B10)	7	2600	37.14	42.5	5.1
(B20)	6	2510	34.05	44.7	5.4
(B30)	7	2400	38.77	46.6	5.6
(R10)	6	2630	33.32	44.49	5.3
(R20)	7	2580	35.53	40.72	5.1
(R30)	8	2400	33.75	37.92	4.9
(H10)	5	3200	35.5	48.9	5.7
(H 20)	6	3100	35.9	46.2	5.4
(H 30)	6	3000	38.16	47.9	5.0
(I 10)	9	2600	32.1	38	4.7
(I 20)	8	2500	28.5	31.52	4.1
(I 30)	8	2430	26.95	30.52	3.8

Adding a metal component to cement improves compressive strength across most additive mixes, except for the I20 and I30 mixes, compared with the control mix. The compressive strength increases at 28 days were recorded as a percentage [18.88%, 25.03%, and 30.35%] for [B10, B20, and B30], [24.45%, 13.9%, and 6.07%], for [R10, R20, and R30], [36.78%, 29.23%, and 33.99] for [H10, H20, and H30], and 6.29 for I10, respectively. However, the I20 and I30 mixes showed reductions in compressive strength of 11.83% and 14.63%, respectively. On the other hand, the tensile strength of the concrete containing additives were increased by [23.9%, 17.4% and 8.7%] for [H10, H20 and H30], [10.9%, 17.4%, and 21.7 %] for [B10, B20, B30], [15.2%, 10.9%, and 6.5%] for [R10, R20, and R30], and 2.2 for I10, respectively. However, the I20 and I30 mixes showed reductions in tensile strength of 10.9% and 17.4%, respectively. Generally, incorporating waste additives with higher specific gravity and fine particles increases concrete density. Their fine particles improve packing density and interfacial bonding by filling the spaces between the cement grains^23^. In addition, these additives enhance the pozzolanic action, thereby strengthening the concrete^10,14^.

### Microstructure analysis

**Fig. 5:**
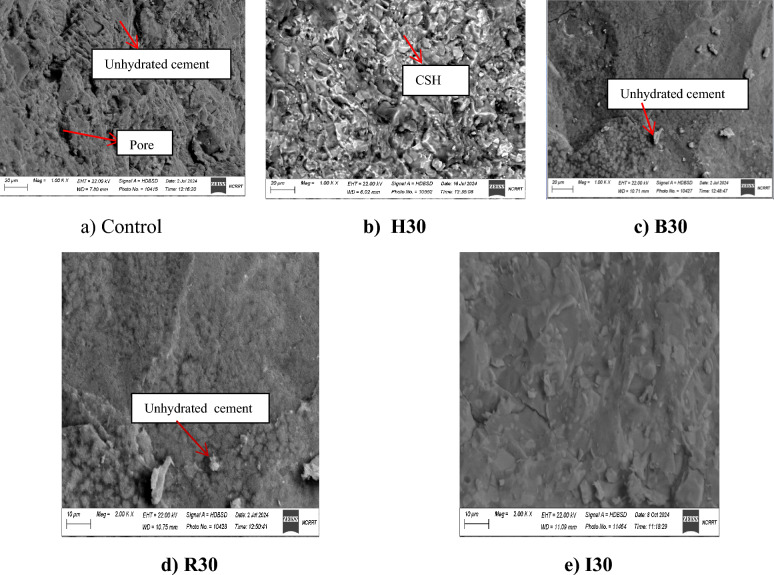
SEM images of control, H30, B30, B30, and I30 mixes.

A comparative analysis was conducted against control samples to investigate the influence of iron waste on concrete morphology. The outcomes are displayed in Fig 5. As shown in Fig 5-a, the control sample contains a considerable amount of unhydrated cement and fewer C-S-H crystal formations. Moreover, its porosity is higher, and its homogeneity is lower than in hematite-bearing samples. In Figure (5-b), it is apparent that waste iron reduces porosity-induced cumulative incursion more effectively than the control sample. The addition of waste iron results in reduced porosity compared to the control sample, likely due to its high specific gravity, which contributes to a denser matrix, as shown in Figures (5-c) and (5-d). In addition, it is notable that incorporating the iron waste makes the sample denser than the control sample, and thus, the initial voids, flaws, and gaps between particles are filled with materials. The microstructure of the concrete containing hematite appeared to be a much denser matrix than the control sample, rolling mill, brake lining, and iron filling samples, which is attributed to the high specific gravity and fineness of hematite particles, which can fill the pores between cementitious material particles in the microstructure and lead to a denser sample as shown in Fig 5-b. This might account for the enhancement of concrete’s mechanical properties due to the inclusion of iron metal (waste iron), as discussed above.

**Fig. 6:**
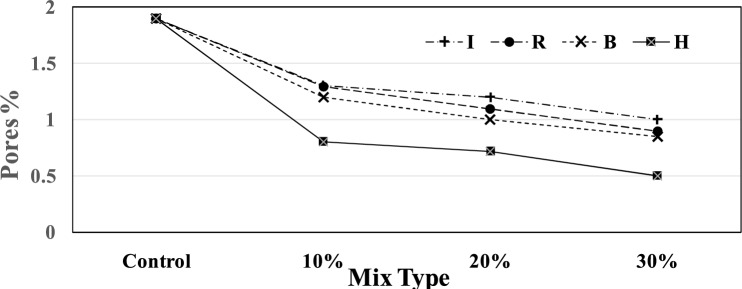
Pore percentages determined by SEM analysis at 28 days for all mixture types.

Fig 6 shows the pores observed in backscattered images processed at 28 days for the control, rolling mill, brake lining, iron filling, and hematite samples. The addition of iron waste led to a noticeable reduction in pore size. These observations agree with the conclusions found in^35^. The percentage decreases were 31.5, 42.5, and 52.6% for the Rolling mill; 36.8, 47.3, and 55.3% for the brake lining; 31.5, 36.8, and 36.8% for the iron filling; and 57.8, 62.1, and 73.6% for the hematite.

### Radiation properties

**Fig. 7:**
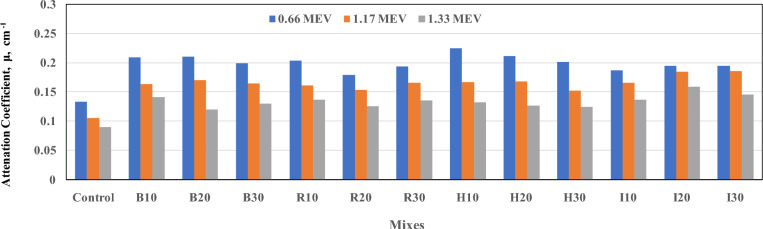
Attenuation coefficient for all mixes for energy 0.66, 1.17, and 1.33 MEV.

The average results from three separate tests conducted at energies of 0.66, 1.17, and 1.33 MeV of gamma attenuation are shown in Fig 7 and Table 4. The results present the LAC, μ (cm⁻^1^), and the HVT and TVT at different energies as a function of concrete density.

**Table 4:** Results of gamma attenuation at different energies.

	Energy	0.66 MEV			1.17 MEV			1.33 MEV		
Mix	Density (kg/m^3^)	Attenuation Coefficient µ cm^-1^	HVT cm	TVT Cm	Attenuation Coefficient µ cm^-1^	HVT cm	TVT cm	Attenuation Coefficient µ cm^-1^	HVT cm	TVT cm
Control	2300	0.1340	5.2164	17.1835	0.1061	6.5881	21.7021	0.0923	7.5731	24.9469
(B10)	2600	0.2095	3.3365	10.9909	0.1640	4.2621	14.0402	0.1421	4.9190	16.2040
(B20)	2510	0.2101	3.3269	10.9595	0.1700	4.1117	13.5447	0.1236	5.6553	18.6294
(B30)	2400	0.1994	3.5055	11.5476	0.1650	4.2363	13.9551	0.1325	5.2754	17.3781
(R10)	2630	0.2038	3.4298	11.2983	0.1620	4.3148	14.2135	0.137	5.1021	16.8072
(R20)	2580	0.1794	3.8963	12.8350	0.1532	4.5626	15.0300	0.1256	5.5652	18.3328
(R30)	2400	0.1935	3.6124	11.8997	0.1658	4.2159	13.8878	0.1358	5.1450	16.9483
(H10)	3200	0.2532	2.7606	9.0939	0.1666	4.1956	13.8211	0.1325	5.2722	17.3676
(H 20)	3100	0.2456	2.8460	9.3754	0.1678	4.1656	13.7222	0.1265	5.5219	18.1900
(H 30)	3000	0.2012	3.4741	11.4443	0.1523	4.5896	15.1188	0.1247	5.6018	18.4532
(I 10)	2600	0.1870	3.7379	12.3133	0.1658	4.2159	13.8878	0.1369	5.1029	16.8097
(I 20)	2500	0.1952	3.5809	11.7961	0.1852	3.7742	12.4330	0.1589	4.3973	14.4854
(I 30)	2430	0.1953	3.5791	11.7900	0.1856	3.7661	12.4062	0.1456	4.7981	15.8056

Fig 7 demonstrates the experimental LAC (µ) values of the prepared samples at energies of 0.66, 1.17, and 1.33 MeV. In general, the results showed that the H-series concrete samples had the highest LAC. This is attributed to the highest hematite density, which reaches 4300 kg/m^3^. On the other hand, the LAC of the studied samples (B & R and I) was higher than that of the control samples due to higher destinies for brake lining, the rolling mill, and filling iron. The overall gain in shielding across mix types was achieved; H mixes gave the highest gains, at (88%, 83%, and 51%) for ratios of 10%, 20%, and 30%, respectively, at an energy of 0.66 MeV. However, the LAC of mixes containing waste iron shows an increase by (56%, 57 %, and 49%) for B mixes of ratio 10%, 20%, and 30%. In the case of R mixes, the increases are by (52%, 34%, and 44%) of 10%, 20%, and 30% comparing to the control mix, respectively. In addition, I mixes have increased LAC by 40%, 46%, and 46% compared with the control mix at 10%, 20%, and 30%, respectively. In addition, the LAC for 1.17 and 1.33 MEV followed a similar trend. It is worth noting that the H mix with 10% showed the highest mechanical and radiation-shielding properties, indicating the optimal mix proportion among all suggested mixes. Therefore, such a mix should be examined from the perspectives of scalability and the practical implementation in real-world construction applications in future studies.

The above results showed a positive contribution of the atomic numbers of the additives used to the concrete’s shielding effect, since hematite has the highest atomic number and a stable composition, thereby increasing density. However, the other samples containing iron waste showed an increase in LAC relative to the control sample; this increase was lower than that observed with hematite due to impurities and voids. All samples exhibited higher LAC than the control sample due to the presence of heavy elements, such as Fe, in hematite and iron waste, which enhance photon absorption and scattering, as mentioned in Ref^23^.

The effectiveness of radiation shielding is commonly assessed using the HVL and TVL parameters^36^, which characterize a material’s attenuation capacity. As the LAC values decreased with increasing γ-ray energy, HVL and TVL increased correspondingly, following an inverse trend (Table 4). This behavior is attributed to the attenuation of radiation within a smaller zone at lower energies. Elabbasy et al^22.^ stated that HVT and TVT values are inversely related to the LAC. As expected, the LAC values for all mixes decreased with increasing γ-ray energy, reflecting the lower probability of photon interactions at higher energies. On the other hand, the decrease in the mass attenuation coefficient (MAC) is attributed to the direct proportionality between LACs and MACs of prepared concretes, where MAC = LAC/ρ; see Ref.^27^.

**Fig. 8:**
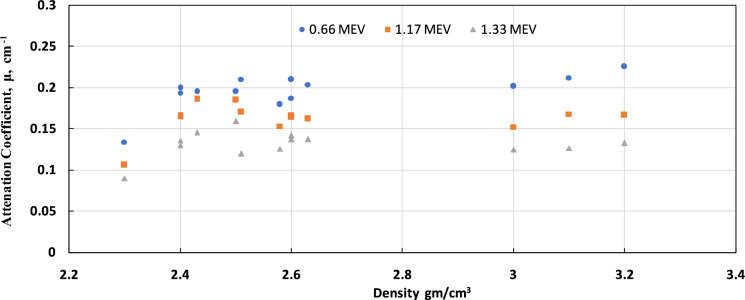
Relation between density and attenuation for all mixes for energies 0.66, 1.17, and 1.33 MEV.

As gamma-ray energy increases, the LAC of concrete decreases. This means that higher-energy gamma rays are less effectively absorbed by concrete than lower-energy gamma rays. Fig 8 shows the relationship between the attenuation coefficient and density for all tested mixtures. The attenuation coefficient may be correlated with concrete density, especially at high densities.

## Conclusion

The goal of this research was to study the effect of adding steel additives (waste materials or steel ore (iron oxide) as a cement replacement on the mechanical and radiation shielding properties of sustainable concrete containing slag as coarse aggregates. From the present experimental results, the following conclusions can be stated

1. In most mixes, the replacement of cement with a steel additive or hematite powder increased mechanical properties. Among all mixes, the 10% hematite blend showed the greatest increase in compressive and tensile strengths by 36.78% and 23.9%, respectively.
2. The microstructure analysis showed that replacing cement with steel additives, such as brake lining, rolling mill scale, iron filings, or hematite concrete, results in denser concrete.
3. SEM image analysis revealed a notable reduction in the percentage of pores in the mixes with steel waste compared to the control mix. The greatest reduction in pores across all mixes was observed when 30% of the cement was replaced with hematite.
4. Based on the gamma attenuation factor results, the highest gain was achieved with hematite mixes, which showed a density increase of approximately 39%.
5. Replacing cement with steel waste/additives reduces the thickness of the shielding concrete and provides the required half-value thickness and attenuation coefficient.

This study is limited to investigating the short-term mechanical properties, microstructure, and radiation properties, without any cost analysis. Therefore, further investigations into the long-term behavior under aggressive environmental conditions, including high-temperature exposure, chemical attack, gamma-ray exposure, and combined loading scenarios, are warranted. Furthermore, future studies should focus on cost analysis and life-cycle assessment of such concretes.

## Data Availability

All data generated or analyzed during this study are included in this article.
